# Supplementary material for: New diagnostic methods for Escherichia marmotae and the first report of its identification in clinical isolates in North America
Source: Front Microbiol. 2025 Sep 5;16:1664775. doi: 10.3389/fmicb.2025.1664775 (PMC12446263; doi:10.3389/fmicb.2025.1664775)
Supplement: Supplementary file 1 [file Table_1.docx]

Title : New Diagnostic Methods for *Escherichia marmotae* and the First Report of its Identification in Clinical Isolates in North America

Pelumi M. Oladipo^1^, Robert J. Tibbetts^2^, Audun Sivertsen^3^, Justin M. Barger^2^, Torbjørn S. Bruvold^3^, Alemu Fite^4^, Matthew Sims^5^, Marcus Zervos^6^, Ali Jomaa^7^, and Jeffrey L. Ram^1,7^

^1^Department of Biochemistry, Microbiology, and Immunology, Wayne State University, Detroit, MI 48201 USA

^2^Department of Microbiology, Henry Ford Health System, Detroit, MI 48202 USA

^3^Department of Microbiology, Haukeland Hospital, Bergen, Norway

^4^Microbiology Laboratory, Corewell Health, Royal Oak, MI USA

^5^William Beaumont University Hospital, Corewell Health, Royal Oak, MI USA

^6^Department of Infectious Diseases, Henry Ford Health System, Detroit, MI 48202 USA

^7^Department of Physiology, Wayne State University, Detroit, MI 48201 USA

Corresponding author: Jeffrey L. Ram [jeffram@wayne.edu](mailto:jeffram@wayne.edu); Pelumi M. Oladipo [hl8030@wayne.edu](mailto:hl8030@wayne.edu)

Table of Contents.

**Table S1: Average Cq threshold cycle number of strains of *E. marmotae*, *E. coli* and *Escherichia* cryptic clades**

**Table S2: MALDI SPECTRA result showing IVD and RUO scores and the specific peak in *E. marmotae,* *E.coli* and other *Escherichia* cryptic clades.**

**Table S3: MALDI SPECTRA result showing IVD and RUO scores and the specific peak in *clinical*  *E.coli* from Henry Ford Laboratory**

**Table S4: Summary of Whole Genome Sequencing Assembly, Completeness and Gene Annotation of *E. marmotae* Clinical Isolate HFH1**

TABLE S5: Complete Virulence Genes list of *E. marmotae* HFH1. See supplemental Excel file.

TABLE S6: Antibiotic Resistance Genes oF *E. marmotae* HFH1. See supplemental Excel file.

**Supplementary Data:**

**Table S1: Average Cq threshold cycle number of strains of *E. marmotae*, *E. coli* and *Escherichia* cryptic clades**

| Strains | *uidA* | *uidB* | *adk* | *lipB* |
| --- | --- | --- | --- | --- |
| C4B | 19.33 | 20.51 | 18.87955 | ND |
| C5 | 19.51 | 20.01 | 18.5445 | ND |
| C6 | 19.14 | 20.41 | 19.10844 | ND |
| C1 | 19.46 | 20.45 | 18.23005 | ND |
| C7 | 20.09 | 20.18 | 18.5719 | ND |
| C8 | 19.17 | 19.89 | 19.68103 | ND |
| AS8 | 19.76 | 20.34 | 20.34157 | ND |
| C9 | 19.69 | 20.06 | 18.39248 | ND |
| C10 | 19.26 | 20.57 | 18.28248 | ND |
| C11 | 19.96 | 20.29 | 19.34203 | ND |
| C12 | 19.61 | 20.73 | 18.34626 | ND |
| C3 | 19.51 | 20.45 | 19.43225 | ND |
| AS14 | 19.79 | 20.27 | 18.30907 | ND |
| AS15 | 19.78 | 20.50 | 18.34307 | ND |
| AS16 | 19.97 | 20.47 | 18.91359 | ND |
| AS17 | 19.56 | 20.65 | 19.74667 | ND |
| AS18 | 19.78 | 20.30 | 18.33043 | ND |
| C2 | 20.07 | 20.26 | 19.37026 | ND |
| C4A | 19.76 | 20.14 | 18.79661 | ND |
| AS21 | 20.15 | 20.56 | 18.56578 | ND |
| AS22 | 19.59 | 20.84 | 19.28115 | ND |
| AS23 | 20.55 | 20.61 | 18.9066 | ND |
| RAM 3024 | 19.51 | 21.31 | 19.42667 | ND |
| RAM 3032 | 19.47 | 20.42 | 19.53464 | ND |
| RAM 3054 | 17.92 | 21.07 | 18.39333 | ND |
| RAM 3050 | 19.08 | 21.90 | 19.11333 | ND |
| TW 09308 | 19.44 | 20.99 | 19.30333 | ND |
| RAM HFH1 | 19.71 | 21.72 | 19.35 | ND |
| E.coli 250 | ND | ND | 21.39044 | 20.18 |
| E.coli 430 | ND | ND | 18.61581 | 20.49 |
| E.coli 1406 | ND | ND | 19.34203 | 19.41 |
| E.coli 970 | ND | ND | 18.01292 | 19.60 |
| TW 15976 | ND | ND | 19.43225 | ND |
| TW 15832 | ND | ND | 18.30907 | ND |
| TW 09254 | ND | ND | 18.00973 | ND |

ND =Not Detected

**Table S2: MALDI SPECTRA result showing IVD and RUO scores and the specific peak in *E. marmotae,* *E.coli* and other *Escherichia* cryptic clades.**

| **Strains** | **IVD score** | **RUO scores** | **Species Specific Peak*** |
| --- | --- | --- | --- |
| TW14263 alias Ram 3024 | 98.3 | 0 | 7261.267 |
| TW14264 alias Ram 3032 | 99.9 | 0 | 7260.751/7261.267 |
| TW14265 alias Ram 3050 | 99.9 | 0 | 7261.260/7260.376 |
| TW14266 alias Ram 3054 | 99.9 | 0 | 7260.878/7261 |
| TW14267 alias Ram 3318 | 99.9 | 77.6 | 7261.133 |
| TW 09308 | 99.9 | 0 | 7261.649 |
| TW 14351 | 99.9 | 0 | 7260 |
| TW 15825 | 99.9 | 0 | 7261.82 |
| TW 15841 | 99.9 | 75.2 | 7259.911 |
| TW 15835 | 99.9 | 84.2 | 7260.802 |
| TW 15848 | 99.9 | 0 | 7260.802 |
| TW 15839 | 99.9 | 0 | 7261.184 |
| TW 15833 | 99.9 | 0 | 7262.075 |
| TW 15846 | 99.9 | 0 | 7261.148 |
| TW 15836 | 99.9 | 0 | 7260.639 |
| TW 15840 | 99.9 | 0 | 7260.511 |
| TW 15834 | 99.9 | 0 | 7260.674 |
| C4B | 99.9 | 0 | 7263.837 |
| C5 | No ID | 0 | 7221.083 |
| C6 | 99.9 | 0 | 7263.964 |
| C1 | NO ID | 0 | 7200.514 |
| C7 | 99.9 | 0 | 7265.111 |
| C8 | 99.9 | 0 | 7264.000 |
| AS8 | NO ID | 0 | 7265.493 |
| C9 | 99.9 | 0 | 7265.621 |
| C10 | 99.9 | 0 | 7265.621 |
| C11 | 99.9 | 0 | 7264.729 |
| C12 | 99.9 | 0 | 7268.298 |
| C3 | 99.9 | 0 | 7266.131 |
| AS14 | 99.9 | 0 | 7201.148 |
| AS15 | 99.9 | 0 | 7201.148 |
| AS16 | 99.9 | 0 | 7266.131 |
| AS17 | 99.9 | 0 | 7262.128 |
| AS18 | 99.9 | 0 | 7261.364 |
| C2 | 99.9 | 0 | 7261.746 |
| C4A | 99.9 | 0 | 7262.128 |
| AS21 | 99.9 | 0 | 7262.765 |
| AS22 | 99.9 | 0 | 7261.874 |
| AS23 | 99.9 | 0 | 7263.529 |
| TW 15976 | 99.9 | 80.0 | 7274.891 |
| TW 15838 | 99.9 | 83.4 | 7274.008 |
| TW 15951 | 99.9 | 99.9 | 7274.381 |
| TW 15832 | 99.9 | 0.00 | 7274.008 |
| TW 09254 | 99.9 | 87.2 | 7273.361 |
| TW 09231 | 99.9 | 87.6 | 7274.253 |
| TW 14182 | 99.9 | 81.2 | 7273.999 |
| TW 11588 | 99.9 | 80.0 | 7273.498 |
| E.coli 250 | 99.9 | 99.0 | 7272.988 |
| E.coli 430 | 99.9 | 99.9 | 7273.370 |
| E.coli 1406 | 99.9 | 98.4 | 7273.106 |
| E.coli 970 | 99.9 | 93.9 | 7273.999 |

*Where the spectrum was determined for both the original Ram Lab strains 3032, 3050, and 3054 and also for the isolates derived from those original strains (TW14264, TW14265, and TW14266, respectively), the values from the independently determined spectral peak are both given. “NO ID” is shown where the BioMérieux instrument was unable to give an IVD identification.

**Table S3: MALDI SPECTRA result showing IVD and RUO scores and the specific peak in *clinical*  *E.coli* from Henry Ford Laboratory**

| Strains | IVD Scores | RUO scores | Peaks |
| --- | --- | --- | --- |
| M43600-1 | 99.9 | 0 | 7218.072 |
| T501076 - 1 | 99.9 | 98.5 | 7270.61 |
| S382911 - 1 | 99.9 | 0 | 7270.816 |
| H594473 - 1 | 99.9 | 0 | 7270.865 |
| S424820 - 1 | 99.9 | 93.5 | 7271.049 |
| X141675 - 2 | 99.9 | 81.5 | 7271.071 |
| F466878 - 1 | 99.9 | 82.5 | 7271.584 |
| W450286-1 | 99.9 | 0 | 7271.601 |
| F434517 - 1 | 99.9 | 90 | 7271.651 |
| M446123 - 1 | 99.9 | 87.5 | 7271.703 |
| H2785 - 1 | 99.9 | 83.3 | 7271.736 |
| W58342 - 1 | 99.9 | 87.2 | 7271.819 |
| F516168 - 1 | 99.9 | 93.8 | 7271.827 |
| X102872 - 1 | 99.9 | 97.8 | 7271.911 |
| T579217 - 1 | 99.9 | 95.2 | 7271.937 |
| H587114 - 2 | 99.9 | 87.3 | 7271.994 |
| T362091-1 | 99.9 | 0 | 7272.026 |
| T483117 | 99.9 | 95.2 | 7272.219 |
| W75726 - 1 | 99.9 | 82.5 | 7272.223 |
| X141675 -1 | 99.9 | 0 | 7272.344 |
| M583411 - 1 | 99.9 | 75.4 | 7272.347 |
| M568608 - 1 | 99.9 | 99.9 | 7272.394 |
| W682619-1 | 99.9 | 77.5 | 7272.42 |
| F351529 - 1 | 99.9 | 89.3 | 7272.745 |
| M377597 - 1 | 99.9 | 86.4 | 7272.901 |
| T261647-1 | 99.9 | 98.4 | 7273.117 |
| X269308-2 | 99.9 | 99.9 | 7273.27 |
| W371120 - 2 | 99.9 | 87.6 | 7273.323 |
| S518994 - 1 | 99.9 | 96.3 | 7273.345 |
| M588863 - 1 | 99.9 | 86.5 | 7273.402 |
| M471433 - 4 | 99.9 | 82.5 | 7273.435 |
| X210193 - 2 | 99.9 | 99.9 | 7273.441 |
| T370915-1 | 99.9 | 87.5 | 7273.518 |
| W41195 - 1 | 99.9 | 86.3 | 7273.544 |
| M542834 - 3 | 99.9 | 0 | 7273.551 |
| H699340 - 1 | 99.9 | 99.9 | 7273.591 |
| M41468-1 | 99.9 | 90.5 | 7273.754 |
| X283079-1 | 99.9 | 99.9 | 7273.758 |
| T94348 - 2 | 99.9 | 99.9 | 7273.829 |
| W689970-1 | 99.9 | 0 | 7273.876 |
| W140653 - 1 | 99.9 | 83.3 | 7273.897 |
| T286241 | 99.9 | 87.2 | 7273.915 |
| H449992-1 | 99.9 | 85 | 7273.962 |
| M62714 | 99.9 | 87.5 | 7273.98 |
| H88796 - 1 | 99.9 | 75.8 | 7273.991 |
| F704616-1 | 99.9 | 90.2 | 7273.994 |
| H567166-1 | 99.9 | 99.9 | 7273.638 |
| H570493-1 | 99.9 | 79.6 | 7275.210 |
| H566692-1 | 99.9 | 93.1 | 7274.405 |
| H566692-2 | 99.9 | 90.2 | 7273.406 |
| H565851-1 | 99.9 | 0 | 7272.648 |
| T482874-1 | 99.9 | 98.4 | 7273.384 |
| H577637-1 | 99.9 | 97.5 | 7272.963 |
| H577637-2 | 99.9 | 99.9 | 7275.343 |
| F270595-1 | 99.9 | 96.7 | 7274.282 |
| H573512-1 | 99.9 | 86.5 | 7274.647 |
| S518900 - 1 | 99.9 | 0 | 7274.009 |
| M61647-1 | 99.9 | 95.4 | 7274.027 |
| S520458 - 1 | 99.9 | 0 | 7274.074 |
| F544368 - 2 | 99.9 | 99.9 | 7274.101 |
| W61945 - 1 | 99.9 | 92.3 | 7274.11 |
| W156575 - 4 | 99.9 | 98.4 | 7274.166 |
| W686297-1 | 99.9 | 83.3 | 7274.176 |
| W674048-3 | 99.9 | 80.1 | 7274.189 |
| H565879-2 | 99.9 | 99.9 | 7274.2 |
| W570445-1 | 99.9 | 92.4 | 7274.232 |
| W443456-2 | 99.9 | 95.5 | 7274.25 |
| W560015-2 | 99.9 | 86.3 | 7274.256 |
| W558193-1 | 99.9 | 84.7 | 7274.283 |
| X688583-1 | 99.9 | 97.6 | 7274.295 |
| H75885 - 1 | 99.9 | 83.4 | 7274.319 |
| T374094-2 | 99.9 | 89.3 | 7274.358 |
| W425622-2 | 99.9 | 0 | 7274.359 |
| F373521 - 3 | 99.9 | 0 | 7274.366 |
| M479312 - 1 | 99.9 | 75.4 | 7274.39 |
| X285519-1 | 99.9 | 85.1 | 7274.396 |
| T629234 - 1 | 99.9 | 93.1 | 7274.419 |
| W164580 - 1 | 99.9 | 85.4 | 7274.443 |
| T485744 | 99.9 | 92.9 | 7274.447 |
| H16219 - 1 | 99.9 | 82.5 | 7274.448 |
| H109142 - 2 | 99.9 | 0 | 7274.456 |
| X138248 - 11 | 99.9 | 83 | 7274.457 |
| M149181-2? | 99.9 | 87.2 | 7274.475 |
| M577899 - 1 | 99.9 | 81.4 | 7274.505 |
| H688852 - 1 | 99.9 | 81.4 | 7274.512 |
| X185929 - 1 | 99.9 | 77.9 | 7274.536 |
| M13245-2 | 99.9 | 99.9 | 7274.544 |
| T361221-2 | 99.9 | 87.2 | 7274.555 |
| M164781-1 | 99.9 | 93 | 7274.591 |
| T260545-1 | 99.9 | 81.4 | 7274.677 |
| F508018 - 1 | 99.9 | 93.5 | 7274.695 |
| M414328 - 1 | 99.9 | 93.1 | 7274.703 |
| W371126 - 1 | 99.9 | 99.9 | 7274.725 |
| M259498-2 | 99.9 | 87.2 | 7274.727 |
| W175456 - 1 | 99.9 | 77.6 | 7274.749 |
| F684244-1 | 99.9 | 90.1 | 7274.763 |
| W138610 - 1 | 99.9 | 75.8 | 7274.777 |
| T491542-1 | 99.9 | 91.9 | 7274.786 |
| T260545-2 | 99.9 | 87.2 | 7274.805 |
| F710862-1 | 99.9 | 87.5 | 7274.806 |
| X155929 - 2 | 99.9 | 99.9 | 7274.812 |
| W174094 - 1 | 99.9 | 83.9 | 7274.825 |
| M164781-2 | 99.9 | 99.9 | 7274.846 |
| M259498-3 | 99.9 | 97.7 | 7274.854 |
| W202658 - 1 | 99.9 | 83.3 | 7274.855 |
| H685711 - 1 | 99.9 | 93.1 | 7274.922 |
| W32749 - 1 | 99.9 | 0 | 7274.951 |
| T273544-1 | 99.9 | 82.4 | 7274.969 |
| H449992-2 | 99.9 | 89.4 | 7274.982 |
| X106451 - 1 | 99.9 | 98.9 | 7274.997 |
| T66906 - 1 | 99.9 | 99.9 | 7275.057 |
| W434641-1 | 99.9 | 99.9 | 7275.09 |
| T620280 - 1 | 99.9 | 84.3 | 7275.093 |
| H568852-1 | 99.9 | 86.4 | 7275.1 |
| F267089-1 | 99.9 | 0 | 7275.108 |
| M381064 - 1 | 99.9 | 87.2 | 7275.162 |
| M506012 - 1 | 99.9 | 90 | 7275.171 |
| W82552 - 1 | 99.9 | 97.5 | 7275.184 |
| S502577 - 3 | 99.9 | 98.5 | 7275.185 |
| W681466 | 99.9 | 84.4 | 7275.209 |
| W463421 | 99.9 | 99.9 | 7275.215 |
| M403605 -2 | 99.9 | 99.9 | 7275.218 |
| M155733-2 | 99.9 | 80.8 | 7275.223 |
| H660901 - 1 | 99.9 | 99.9 | 7275.234 |
| H565879-1 | 99.9 | 99.9 | 7275.276 |
| W443191-1 | 99.9 | 98.4 | 7275.302 |
| X688015-9 | 99.9 | 90 | 7275.303 |
| T356776-3 | 99.9 | 87.5 | 7275.322 |
| S514130 - 1 | 99.9 | 81.3 | 7275.327 |
| W460868-2 | 99.9 | 99.9 | 7275.333 |
| W384925 - 1 | 99.9 | 93.7 | 7275.356 |
| M362604 - 1 | 99.9 | 99.9 | 7275.448 |
| W683804 | 99.9 | 83.7 | 7275.464 |
| W595366-1 | 99.9 | 79.3 | 7275.469 |
| H146023 - 2 | 99.9 | 99.9 | 7275.515 |
| H263911 - 1 | 99.9 | 80.3 | 7275.553 |
| S397665 - 1 | 99.9 | 83.2 | 7275.576 |
| M546434 - 1 | 99.9 | 0 | 7275.587 |
| W681465 | 99.9 | 78.4 | 7275.591 |
| X215180 - 1 | 99.9 | 90.9 | 7275.608 |
| W450382-1 | 99.9 | 0 | 7275.679 |
| T581445 - 1 | 99.9 | 95.3 | 7275.703 |
| T356776-4 | 99.9 | 86.4 | 7275.705 |
| T367996-1 | 99.9 | 0 | 7275.727 |
| H660901 - 2 | 99.9 | 80 | 7275.728 |
| W200381 - 1 | 99.9 | 99.9 | 7275.743 |
| H650703 - 2 | 99.9 | 88.6 | 7275.831 |
| T497129 | 99.9 | 87.2 | 7275.915 |
| T620280 - 6 | 99.9 | 92.4 | 7275.923 |
| T674672 - 1 | 99.9 | 84.3 | 7275.924 |
| M507603 - 1 | 99.9 | 95.4 | 7275.989 |
| T484107 | 99.9 | 84.5 | 7276.08 |
| T361224-1 | 99.9 | 0 | 7276.084 |
| M152234-1 | 99.9 | 0 | 7276.096 |
| T274016-1 | 99.9 | 89.3 | 7276.11 |
| F518804 - 1 | 99.9 | 99.9 | 7276.346 |
| H29738 - 1 | 99.9 | 89.4 | 7276.394 |
| T696469 - 1 | 99.9 | 84.1 | 7276.44 |
| H650703 - 1 | 99.9 | 84.6 | 7276.468 |
| F334315 - 2 | 99.9 | 78 | 7276.637 |
| H650703 - 3 | 99.9 | 90.5 | 7276.851 |
| W682619-2 | 99.9 | 0 | 7276.878 |
| S452562 - 1 | 99.9 | 95.3 | 7276.957 |
| M365126 - 2 | 99.9 | 95.4 | 7277.175 |
| F640317 - 1 | 99.9 | 85.1 | 7277.244 |
| M503941 - 1 | 99.9 | 99.9 | 7277.652 |
| T303591-2 | 99.9 | 99.9 | 7277.669 |
| S389864 - 1 | 99.9 | 87 | 7277.673 |
| M150120-1 | 99.9 | 99.9 | 7277.881 |
| M60781-1 | 99.9 | 92.1 | 7278.279 |
| H263748 - 1 | 99.9 | 77.5 | 7281.616 |
| M152848-1 | 99.9 | 0 | 7281.96 |
| H687065 - 2 | 99.9 | 0 | 7285.062 |
| W681467 | 99.9 | 0 | 7215.946 |
| M325108-1 | 99.9 | 0 | 7217.761 |
| RAM HFH1 | 99.1 | 0 | 7261.439 |

Table S4: Summary of the *Escherichia* strains from GenBank used in *In Silico* analysis using pubMLST.

| S/N | Strains | GenBank Accessions |
| --- | --- | --- |
| 1 | *E. marmotae* RAM 3024 | *JBNVMX000000000* |
| 2 | *E. marmotae* RAM 3032 | *JBNVMU000000000* |
| 3 | *E. marmotae RAM 3050* | *JBNVMV000000000* |
| 4 | *E. marmotae* RAM 3054 | *JBNVMW000000000* |
| 5 | *E. marmotae* RAM 3318 | *JBNVMY000000000* |
| 6 | *E. marmotae* 09308 | *NZ_AEME01000001* |
| 7 | *Escherichia marmotae* 21-MO00613 | JAHCSB000000000 |
| 8 | *Escherichia marmotae* HT073016 | JNBP00000000 |
| 9 | Escherichia coli strain RHB04-C17 | CP058009 |
| 10 | *Escherichia coli* str. K-12 substr. MG1655 | CP009685 |
| 11 | *Escherichia coli* O157:H7 str. 2011EL-1107 | JHLK00000000 |
| 12 | *Escherichia coli* O86 strain JE86-ST05 | BHVN01000001 |
| 13 | Escherichia coli strain UPEC ECZMYU21 ECZMYU | JBBHLN01000000 |
| 14 | *Escherichia coli strain EHEC19* | SSJJ0100000 |
| 15 | *Escherichia coli strain NMEC15* | JAHTGQ010000001.1 |
| 16 | *Escherichia coli 2015C-3820* | ABAEBA000000000.1 |
| 17 | *Escherichia coli AA002* | DADXYW000000000.1 |
| 18 | Escherichia coli AZ-TG73415 | ABAEBU010000000 |
| 19 | *Escherichia coli* PNUSAE000546 | ABAECF000000000.1 |
| 20 | *Escherichia sp*. TW15838 | AEJX00000000 |
| 21 | *Escherichia whittamii AGR6438* | NZ_JAVGZX000000000.1 |
| 22 | *Escherichia whittamii C2-3* | NZ_JAINCF000000000.1 |
| 23 | Escherichia whittamii Ngtc RS 24 | NZ_JAMWXD000000000.1 |
| 24 | *Escherichia whittamii* AGR6436 | NZ_JAVGZY000000000. |
| 25 | *Escherichia whittamii* Sa2BVA5 | JACSQ1000000000 |
| 26 | *Escherichia ruyisiae* TW09276 | AEJV00000000 |
| 27 | *Escherichia ruyisiae* AGR4808 | JAVGQM000000000 |
| 28 | *Escherichia ruyisiae* S1-IND | CP112984 |
| 29 | *Escherichia ruyisiae* TW11588 | AEMF00000000 |
| 30 | *Escherichia ruyisiae* C15-8 | \| JAINAA000000000 \| \| --- \| |
| 31 | *Escherichia ruyisiae* C18-2 | JAIMZW000000000 |
| 32 | *Escherichia ruyisiae* TW14182 | AEJZ00000000 |

**Table S5: Summary of Whole Genome Sequencing Assembly, Completeness and Gene Annotation of *E. marmotae* Clinical Isolate HFH1**

| Parameters* | Data |
| --- | --- |
| Assembly | |
| Number of Contigs | 74 |
| Total length (bp) | 4614133 |
| GC Content (%) | 50.37 |
| N50 **(**bp**)^a^** | 177349 |
| N75**(**bp**)^a^** | 96904 |
| L50 **(**bp**)^a^** | 8 |
| L75**(**bp**)^a^** | 16 |
| Annotation of the Genome | |
| Total number of Genes | 4353 |

**^a^**The meanings of the listed Parameters are defined by the Quality Assessment Tool (QUAST) (Gurevich et al., 2013)) in which N50 and N75 are the lengths for which the collection of all contigs of that length or longer covers at least 50% or 75%, respectively, of the assembly, and L50 and L75 are the number of contigs equal to or longer than N50 and N75, respectively. In other words, L50 is the minimal number of contigs that cover half the assembly.

TABLE S6: Complete Virulence Genes list of *E. marmotae* HFH1. See supplemental Excel file.

TABLE S7: Antibiotic Resistance Genes of *E. marmotae* HFH1. See supplemental Excel file.
